# Modifier Effects of APOE E2 and E4 Alleles on the age at onset of Alzheimer Disease in Puerto Rican G206A Mutation Carriers: A Family‐Based Study

**DOI:** 10.1002/alz.092650

**Published:** 2025-01-09

**Authors:** Balaji Kannappan, Rong Cheng, Tu Nguyen, Ivonne Z. Jimenez‐Velazquez, Angel Piriz, Aleyda Maldonado, Badri N. Vardarajan, Dolly Reyes‐Dumeyer, Rafael A. Lantigua, Zhezhen Jin, Richard Mayeux, Joseph H. Lee

**Affiliations:** ^1^ The Taub Institute for Research on Alzheimer’s Disease and the Aging Brain, Vagelos College of Physicians & Surgeons, Columbia University, New York, NY USA; ^2^ Gertrude H. Sergievsky Center, Vagelos College of Physicians & Surgeons, Columbia University, New York, NY USA; ^3^ Department of Neurology, Vagelos College of Physicians & Surgeons, Columbia University, New York, NY USA; ^4^ Department of Epidemiology, Mailman School of Public Health, Columbia University, New York, NY USA; ^5^ University of Puerto Rico, School of Medicine, San Juan Puerto Rico; ^6^ The Gertrude H. Sergievsky Center, College of Physicians and Surgeons, Columbia University, New York, NY USA; ^7^ Department of Medicine, Vagelos College of Physicians & Surgeons, Columbia University, New York, NY USA; ^8^ Department of Biostatistics, Mailman School of Public Health, Columbia University, New York, NY USA

## Abstract

**Background:**

APOE E4 (APOE4 henceforth) is recognized as a major genetic risk factor for late‐onset Alzheimer's disease (AD), with its impact varying across ethnic subgroups. We investigate the risk of APOE4 and the protective effect of APOE E2 (APOE2 henceforth) in individuals carrying the high‐risk G206A mutation in the PSEN1 gene (G206A henceforth), known to cause early‐onset AD. Despite the G206A mutation's strong association with AD, there is noticeable variability in age at onset among carriers. We aim to elucidate the modifying effects of APOE2 and APOE4 alleles on age at onset for AD among G206A carriers and non‐carriers, accounting for known confounders and their genetic background differences.

**Method:**

This study examined the modifying effects of APOE2 and APOE4 on the risk of AD onset between non‐carriers and carriers of the G206A mutation using multivariable Cox proportional hazards models, examining 505 individuals from 87 Puerto Rican families. We further examined whether APOE2 or APOE4 behave differently in the 2 ethnic subgroups: the Hispanic Whites (H‐White) vs. Hispanic Non‐Whites (H‐Non‐Whites).

**Result:**

As expected, the G206A consistently showed a significant increase in AD risk (HR=10.61, p=9.60E‐30). When APOE4 was examined together with the G206A, it weakly increased the risk (HR=1.72, p=1.81E‐3), while APOE2 showed a weak protective effect but was not significant (HR=0.83, p=0.53). We then examined the APOE effects in ethnic subgroups. Given the small sample size in the H‐Non‐Whites, we compared the mean age at AD onset rather than computing HR. For H‐Whites carrying both G206A‐APOE2, the average age at onset was 60.3 years. Among H‐Non‐Whites, there was only one G206A‐APOE2 carrier, and this person remained unaffected until 87.5 years. Although based on a single case, this significant delay in AD onset signals a potentially critical area for future research in understanding the mechanisms behind the APOE2 protective effect.

**Conclusion:**

The APOE2 allele shows protective effects on AD onset in G206A carriers, specifically in H‐Non‐Whites. A single case of late‐onset in a H‐Non‐White G206A‐APOE2 carrier highlights the need for in‐depth research into APOE2's role across ethnicities. G206A notably heightens the risk of early AD onset, with APOE4 contributing moderately.

